# Identifying the combinatorial control of signal-dependent transcription factors

**DOI:** 10.1371/journal.pcbi.1009095

**Published:** 2021-06-24

**Authors:** Ning Wang, Diane Lefaudeux, Anup Mazumder, Jingyi Jessica Li, Alexander Hoffmann

**Affiliations:** 1 Institute for Quantitative and Computational Biosciences (QCBio), University of California, Los Angeles, California, United States of America; 2 Department of Microbiology, Immunology, and Molecular Genetics, University of California, Los Angeles, California, United States of America; 3 Interdepartmental Program in Bioinformatics, University of California, Los Angeles, California, United States of America; 4 Department of Statistics, University of California, Los Angeles, California, United States of America; University of Wisconsin, Madison, UNITED STATES

## Abstract

The effectiveness of immune responses depends on the precision of stimulus-responsive gene expression programs. Cells specify which genes to express by activating stimulus-specific combinations of stimulus-induced transcription factors (TFs). Their activities are decoded by a gene regulatory strategy (GRS) associated with each response gene. Here, we examined whether the GRSs of target genes may be inferred from stimulus-response (input-output) datasets, which remains an unresolved model-identifiability challenge. We developed a mechanistic modeling framework and computational workflow to determine the identifiability of all possible combinations of synergistic (AND) or non-synergistic (OR) GRSs involving three transcription factors. Considering different sets of perturbations for stimulus-response studies, we found that two thirds of GRSs are easily distinguishable but that substantially more quantitative data is required to distinguish the remaining third. To enhance the accuracy of the inference with timecourse experimental data, we developed an advanced error model that avoids error overestimates by distinguishing between value and temporal error. Incorporating this error model into a Bayesian framework, we show that GRS models can be identified for individual genes by considering multiple datasets. Our analysis rationalizes the allocation of experimental resources by identifying most informative TF stimulation conditions. Applying this computational workflow to experimental data of immune response genes in macrophages, we found that a much greater fraction of genes are combinatorially controlled than previously reported by considering compensation among transcription factors. Specifically, we revealed that a group of known NFκB target genes may also be regulated by IRF3, which is supported by chromatin immuno-precipitation analysis. Our study provides a computational workflow for designing and interpreting stimulus-response gene expression studies to identify underlying gene regulatory strategies and further a mechanistic understanding.

## Author summary

Cells need to sense environmental cues and respond appropriately. One important notion is that different stimuli activate different combinations of transcription factors and that responsive genes are regulated by distinct subsets of these. However, identifying the regulatory strategies by which genes interpret transcription factor activities remains a largely unsolved challenge. In this work we address the question: to what extent are combinatorial transcription factor regulatory strategies identifiable from stimulus-response (input-output) datasets? We present a computational framework to determine the identifiability of gene regulatory strategies, and examine how reliable and quantitative model inference is a function of the quality and quantity of available data. We present an error model that more precisely quantifies uncertainty for perturbation-timecourse data sets by also considering error in the time domain, and achieves an improved performance in identifying and quantifying gene regulatory strategies. With these tools, we generate guidelines for experimental design that optimize limited resources for generating data for model inference. Finally, we apply the workflow to immune response datasets and uncover evidence that many more genes are subject to combinatorial control than previously thought; we offer physical transcription factor binding data to support this finding for one particular group of genes. This demonstrates that the computational workflow may guide studies of the regulatory strategies that govern stimulus-responsive gene expression programs.

## Introduction

A primary goal of biology is to understand biological phenomena in terms of the underlying factors, whether these are cells, molecules or genes. These factors form dynamic regulatory networks whose emergent properties are responsible for biological phenomena. Hence, the systems biology approach employs mathematical models that represent or abstract these networks to interpret experimental data.

For studies of how genes are expressed, the advent of experimental assays that are capable of producing genome-wide measurements of mRNA abundance, chromatin-bound factors and modifications has been revolutionary. A variety of computational approaches have been developed to construct correlations networks based on these large datasets [1–6]; these infer correlative connections between regulators that may reflect a direct or indirect causal relationship, a common cause, or mere coincidence. Because correlative approaches often leverage the statistical power of multiple datapoints from similarly expressed genes, they are not well suited in addressing the regulatory precision of individual genes [7]. That is an important limitation, as many pathological conditions can in fact be traced to a single gene culprit, or a handful [8].

In order to leverage the wealth of genome-wide gene expression datasets for developing a mechanistic understanding of gene expression, prior studies have employed mathematical models that represent the functional interactions between the gene and the key transcription factor (TF) [9–13]. In pioneering work, kinetic models with logic gates were applied to time series data to infer genome-wide transcriptional regulatory networks [6], but questions about the goodness of fit, uniqueness of the solution, and model identifiability remained to be addressed. Simple mathematical models do not describe the detailed gene regulatory mechanisms in the nucleus, that may contain hundreds of factors along large stretches of regulatory DNA, but constitute functional abstractions that may be termed the gene regulatory strategies (GRSs). Thus, GRS models describe functional relationships in a mechanistic modeling framework.

Mathematical models of such GRSs involving a single TF have been successfully fit to datasets from individual mammalian genes, when the TF activity may be induced by a stimulus to provide a perturbation with a defined starting timepoint. In this case both TF activity and target gene expression were measured in a timecourse to provide the data for GRS model fitting. Indeed, for immune response genes this approach allowed GRS model parameters to be fit [10,13], or the GRS model topology to be selected from two alternatives [9]. Such experimentally validated GRS models may then guide the development of finer-grained GRN models that more faithfully describe the physical interactions that give rise to the regulatory strategies.

However, many mammalian genes are not regulated by a single TF, but multiple TFs. TFs collaborate either by compensating for or enhancing each other’s activities. Characterizing what is termed the “logic” of collaborative TFs regulating genes is critical to understanding how the genome is expressed. To quantitatively capture combinatorial gene regulation by multiple TFs, thermodynamic formulations of Boolean AND- and OR-gate-like relationships may be employed to describe molecular interactions between DNA, TFs, and the polymerase-containing transcription machinery that regulates transcription initiation [14–17]. However, identifying the right model for a specific experimental dataset means that not only the most appropriate model topology (e.g. logic gates) needs to be selected to fit the data, but this should be done by considering a range of parameter values (e.g. TF regulation strengths) that should be optimally fit to the data.

A recent study aimed to identify GRSs with combinatorial TF logics using mammalian immune response datasets [18]. To render the model identifiability challenge more tractable, the complexity of matching combinatorial TF models had to be dramatically reduced by two simplifying measures: first, the model parameters were fixed, reducing the task to identify the best model to match the data; and second, models were matched to the mean expression of co-clustered genes, rather than individual genes, thereby avoiding the confounding effects of technical inaccuracy. This second simplification, however, diminishes the rich diversity of regulatory strategies that occur even within co-clustered genes [19,20]. In sum, we conclude that 1) model selection from a library of GRS topologies and quantitative fitting from a range of parameter values must be done at single gene resolution, and 2) this task poses an as yet unresolved model identifiability challenge.

In this study, we have addressed the GRS model identifiability challenge in three steps. First, we systematically delineated GRS model identifiability, thereby identifying GRS models that are easily distinguished from each other, and others that require substantially more data. Second, we developed a Bayesian computational workflow–including a new error model–to use experimental input-output datasets for GRS model selection and parameter fitting. Third, we applied the computational workflow to newly assembled experimental innate immune datasets. Our results demonstrate the utility of the newly developed computational workflow by applying it to an innate immune gene dataset to reveal potentially combinatorially controlled genes in line with reports in other biological systems [21,22].

## Results

### Assessing the distinguishability of gene expression patterns produced by distinct GRSs involving three TFs

Upon cellular stimulation, the activities of stimulus-induced TFs are activated and–in a combinatorial manner–enhance transcription initiation of specific genes. To explore the identifiability of distinct GRS models involving TF combinations, we first examined via a systematic model-based analysis how distinguishable the associated gene expression patterns really are when different TF combinations are activated.

**Fig 1:**
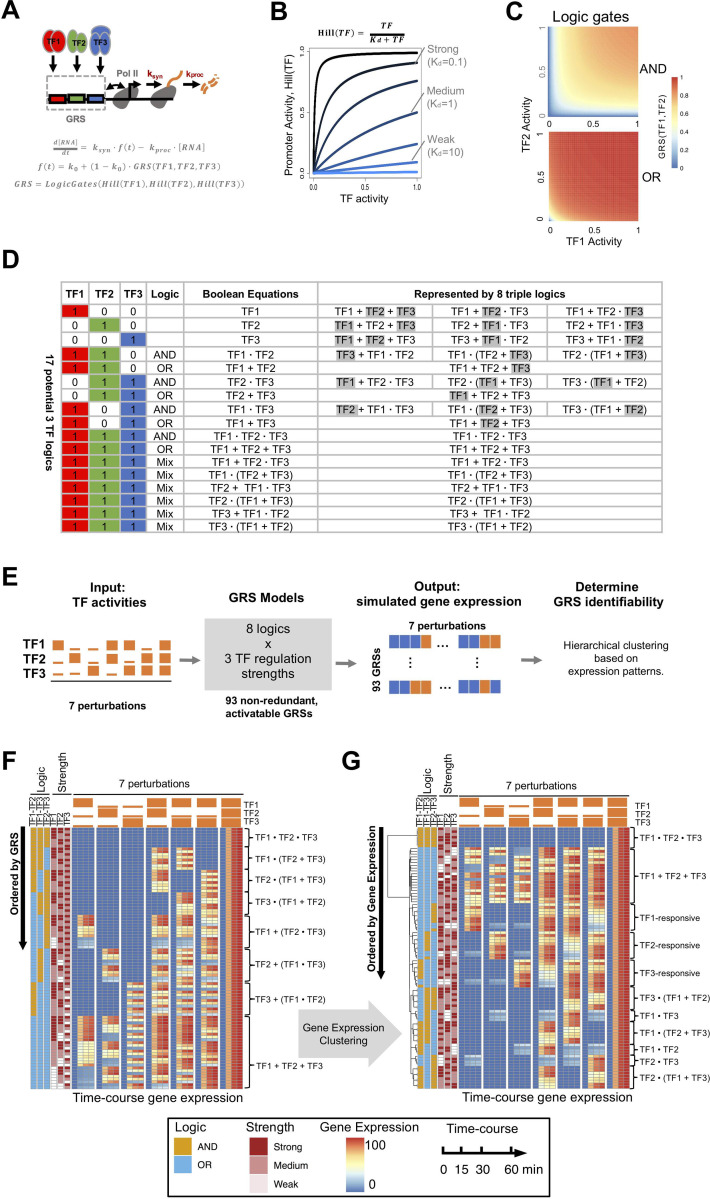
Studying GRSs with stimulus-induced TFs. (A) Schematic of the gene regulation model: stimulus-induced TFs bind to target DNA to induce Pol II-mediated mRNA synthesis, which is followed by its processing and release from the chromatin. Nascent mRNA abundance can be described by a single ordinary differential equation (ODE). Promoter activity is described by thermodynamic models involving Hill functions. (B) Line graphs of promoter activity as a function of a single TF activity depends on the regulation strength (here indicated by 1/K_d_, in black-to-blue scale). We define the regulation strengths as Strong (K_d_ = 0.1), Medium (K_d_ = 1.0), or Weak (K_d_ = 10), as marked in the plot. (C) Heatmaps of promoter activity as a function of logic gates (AND, OR) with varying TF1, TF2 activities as input both with Strong regulation (K_d1_ = K_d2_ = 0.1). (D) Enumeration of all 17 possible AND and OR logic combinations by three TFs. These may be represented by 8 triple logics when single and dual TF logics contain null regulation strengths (*K*_*d*_ ≫ 1) for one or two TFs (marked with grey shading). AND and OR gates are denoted with “∙” and “+” in Boolean algebra. (E) Schematic of the analysis workflow using a set of 7 perturbations with high and low TF activities, to probe 93 non-redundant, activatable GRSs (see S2 Fig), by simulating their gene expression patterns and examining those patterns by hierarchical clustering. (F) Heatmap of gene expression at 0, 15, 30, 60 min from all 93 GRSs in response to a set of 7 perturbation conditions involving 3 TFs. Here the heatmap is ordered by GRSs, their regulatory logic and TF regulation strengths (left columns). (G) The data of panel (F) with GRSs are ordered by hierarchical clustering (single linkage approach) of gene expression using the squared Euclidean distance depicted by the tree. This analysis shows that distinct combinatorial logics may give similar gene expression patterns, and that tripe AND gates with distinct regulation strengths cannot be distinguished with 7 perturbations.

We employed an established model formalism in which messenger RNA abundance of an expressed gene is controlled by synthesis and decay [23], in our case we model nascent mRNA such that the decay term corresponds biologically to its processing and release from the chromatin. An ordinary differential equation models RNA abundance dynamics (Fig 1A), where the processing/chromatin release rate (*k*_*proc*_) is first order and RNA synthesis is zeroth order but is modeled by multiplying the RNA synthesis rate constant (*k*_*syn*_) with the fractional promoter activity (*f (t)*). Fractional promoter activity was modeled by an established thermodynamic formulation with a Hill function that captures the TF regulation strength [17,18], as a function of a disassociation constant (*K*_*d*_). Strong (*K*_*d*_ = 0.1) and Weak (*K*_*d*_ = 10) regulation strengths were defined as the readily saturated and always linear dose-response relationships, respectively, with Medium regulation strength being in-between (Fig 1B). Here we assume no cooperativity in the activation by a single TF (*n*_*H*_ = 1), in line with a previous report [24] (though future work may consider non-linear dose response curves that may have been reported in other systems. Similar thermodynamic formulations [15,17,18] are used to model AND and OR logic gates which consider synergistic or non-synergistic functions between two TFs (Figs 1C and S1, see Methods). We enumerated all possible logics composed by single, dual and triple TFs with AND and OR logic gates (Fig 1D). Single and dual logics can be represented by the same 8 triple logic gates when allowing for null regulation strengths for one or two TFs (see Methods). Thus, only 8 triple logic gates are needed to study all logics formed by the combination of a one, two or three TFs.

To conduct a systematic analysis of GRS identifiability, we enumerated the GRS models (S2 Fig) that cover not only all combinations of AND and OR logics but also 3 TF regulation strengths (*K*_*d*_ = 0.1, 1, 10, referred to as Strong, Medium, Weak) and a fixed pre-mRNA chromatin release and processing rate. Of the possible 216 GRSs, 69 GRSs were removed because they cannot be efficiently activated (due to weak TF regulation strengths), and 54 GRSs were removed because they were logically equivalent with other, resulting in a list of 93 unique, potentially identifiable GRSs. To probe these GRSs, we first considered a set of 7 perturbations involving all combinations of high and low activities induced for each of the 3 TF (2^3^–1 = 7, omitting the case of all three TFs being low) (Fig 1E). We simulated gene expression patterns across timepoints for all 93 GRSs (Fig 1F, Methods), and reordered GRSs based on hierarchical clustering (single linkage method) of their gene expression timecourses, using the squared Euclidean distance across all time points and perturbation conditions as the distance metric between them (Fig 1G). This revealed that some distinct logics produce similar gene expression patterns as they were co-clustered. For example, within the TF1-response cluster we find both the TF1 or (TF2 and TF3) GRS and the triple OR gate GRSs, but what all have in common is that TF1 has strong (S) or medium (M) regulation strength. We next examined the tree height for each cluster. It is apparent that the GRSs within the triple OR gate cluster are easiest to distinguish, and the GRSs within the triple AND gate cluster are hardest to distinguish (indistinguishable with the present 7 perturbations). All the results have been further confirmed with different hierarchical clustering methods (average and complete linkages, S3A Fig).

**Fig 2:**
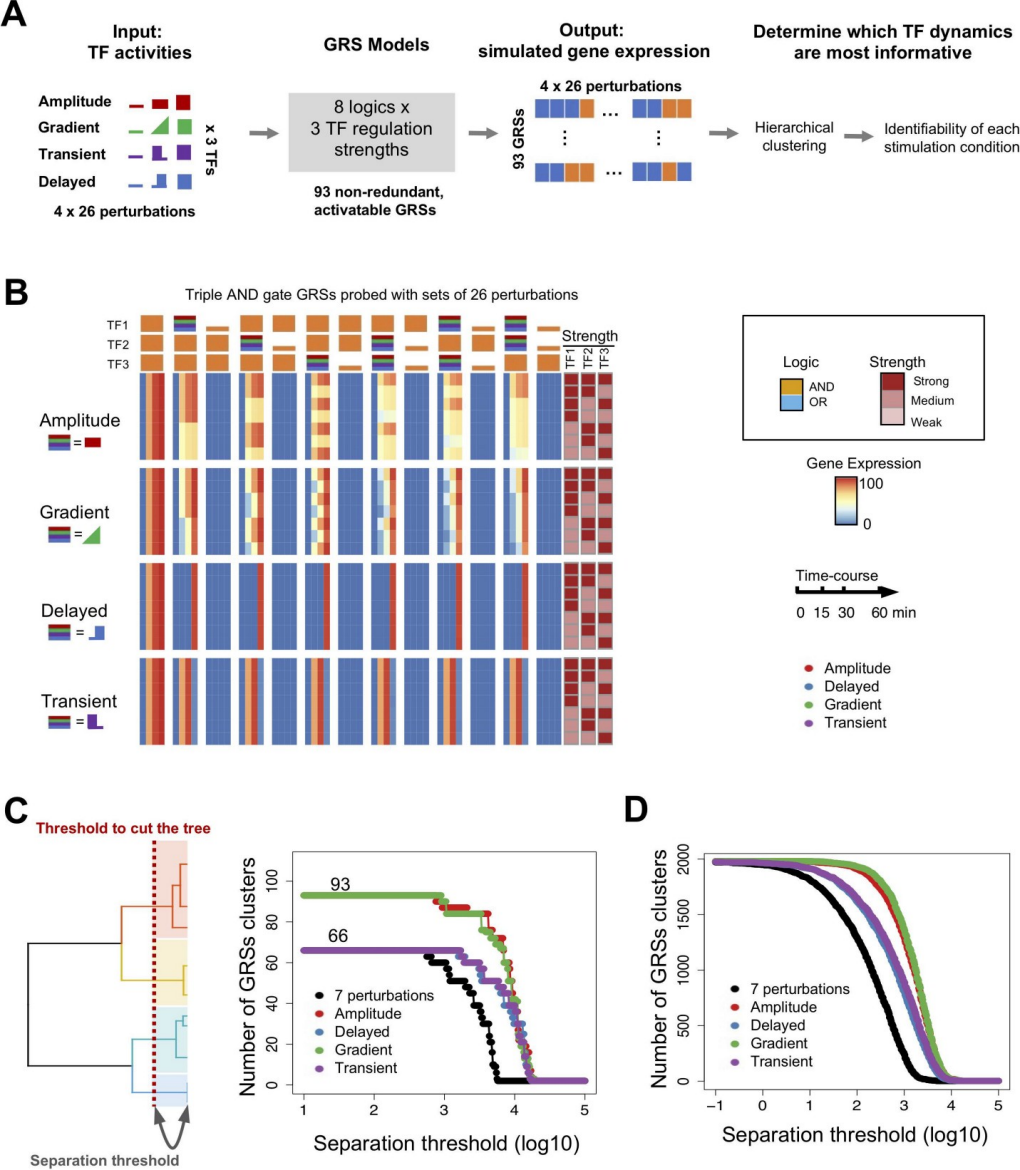
The distinguishability of GRSs depends on the available set of TF perturbations. (A) Schematic of the analysis workflow using 4 sets of 26 perturbations each with amplitude, gradient, transient, and delayed TF dynamics to produce combinatorial TF activities to probe 93 non-redundant, activatable GRSs, by simulating their gene expression patterns and examining those patterns by hierarchical clustering. (B) Heatmap of the triple AND gate GRSs (top 7 rows in (C), probed with one of the 4 sets of 26 perturbations (shown in (B)), containing amplitude modulated TF activities (amplitude, gradient) and temporally modulated TF activities (delayed, transient). The results show that amplitude modulated TF activities best suited to distinguish GRSs are (C) Number of distinguishable GRS clusters as a function of the separation threshold with indicated sets of perturbation combinations. (D) The same plot as panel C but from 1981 inducible GRSs generated by random sampling of 1000 parameter sets for each logic. All 1981 GRS are identified at the lowest separation threshold (-1) for amplitude and gradient perturbations, whereas 1971, 1975, 1976 GRS clusters are identified for high/low, transient, and delayed perturbations, respectively.

As 7 perturbations do not enable distinction of all GRSs, we next explored more diverse perturbation conditions such as transient, delayed, gradient, or intermediate amplitude TF activations (Fig 2A). Examining the triple AND gate GRSs that could not be distinguished with the set of 7 perturbations, we found dramatic differences: amplitude and gradient perturbations readily distinguished these 7 GRSs, but transient and delayed TF temporal dynamics did not (Fig 2B). To summarize, we found that the regulation strength of a TF may be identifiable when perturbation data with TF activities of differential amplitudes is available.

When fitting the GRS models to experimental data, we may expect that lower-quality data may affect the identifiability of less well-separated GRSs more than of widely separated GRSs. To explore how the resolution of each GRS and data quality affects GRS identifiability, we cut the tree at various height thresholds, referred to as separation thresholds, and we determined the number of distinguishable GRS clusters as a function of this threshold with multiple perturbation conditions (Fig 2C). This analysis further confirmed our previous observations: the transient and delayed perturbation conditions only marginally increased GRS identifiability, whereas the amplitude and gradient modulation conditions increased GRS identifiability substantially to distinguishing all 93 GRSs. Moreover, these results were confirmed with alternative hierarchical clustering methods (S3B Fig). The hierarchical tree analysis therefore provides guidance on which GRSs are more likely confused, and indicate which GRSs that are functionally similar to the identified GRS of interest.

The initial study of 93 representative GRSs allowed us to efficiently and systematically reveal the key properties of GRSs with multiple perturbation conditions. We next generalized our results by random sampling of 1000 parameter sets for each logic gate (see Methods). We stringently represented single, dual logics with null regulation strengths of triple logic gates, and sampled all parameters (3 *K*_*d*_, *k*_*syn*_, *k*_*proc*_, *k*_*0*_) from the full parameter space. We found that clusters formed (S3C Fig) that were similar to those observed in the analysis of representative GRSs. We next compared multiple perturbation conditions, and confirmed the result: only amplitude and gradient modulation conditions enabled distinguishing all GRSs (Fig 2D).

### An error model to quantify data uncertainty in value and time

**Fig 3:**
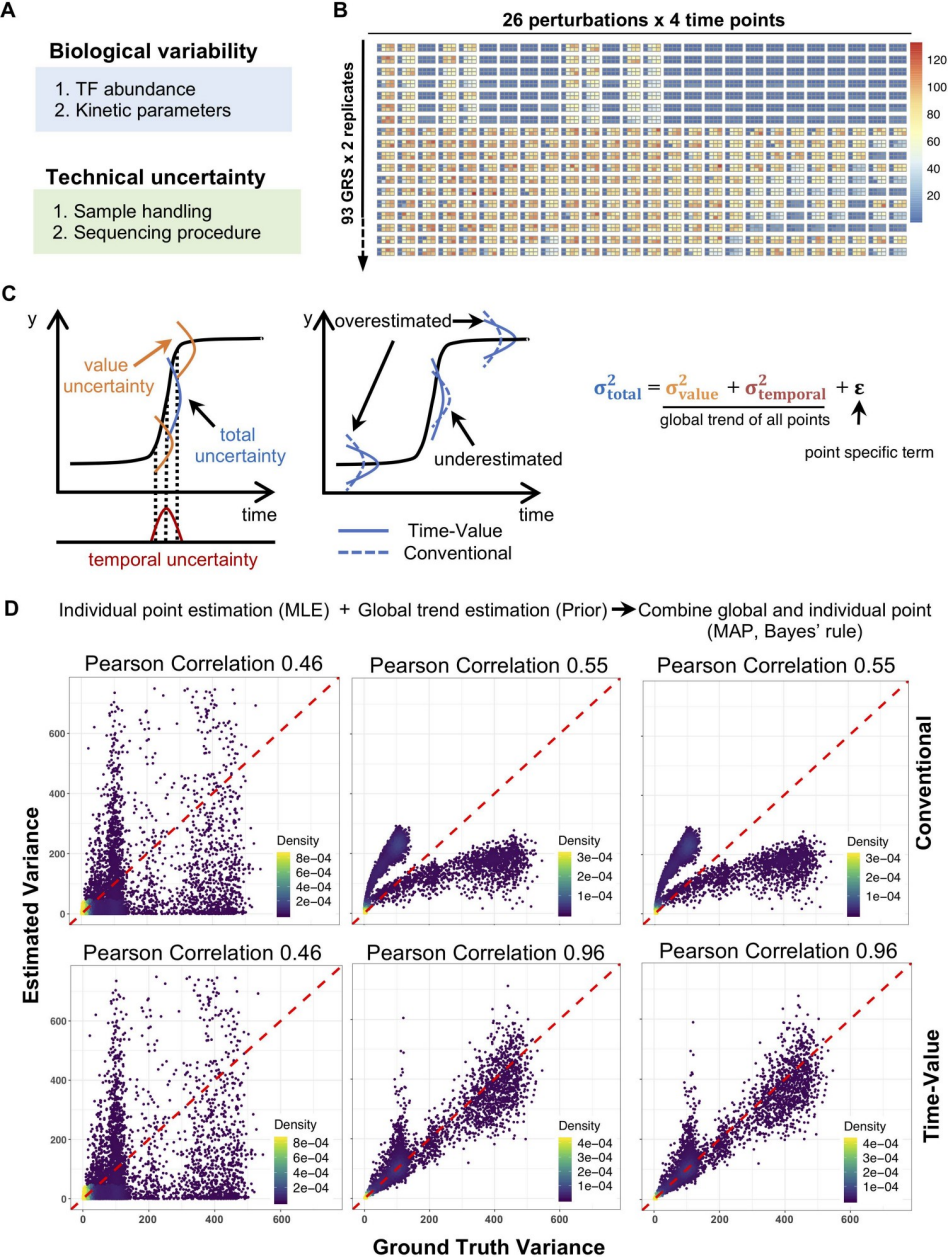
An error model for stimulus-response data that leverages timecourse information to avoid under and over-estimates of measurement uncertainty. (A) Uncertainty sources in data simulation. We considered both biological variability and technical uncertainty. (B) Simulated gene expression data with uncertainty (see Methods). Replicate datasets for 18 of the 93 GRSs are shown here. (C) Diagram of the time-value error model. Left panel shows how observed uncertainty can be decomposed into value and temporal uncertainties. Middle panel shows how the conventional model will under- and over-estimate data uncertainty. Right panel shows that error is decomposed into value uncertainty, temporal uncertainty, and point-specific uncertainty. (D) Uncertainty estimation with the temporal-value and conventional error model. For the left column, we estimated data uncertainty from each point using Maximum Likelihood Estimation (MLE) (98% points are shown). For the middle column, we estimated the global trend as prior. By applying Bayes’ rule, we obtained posterior estimates, right column. Pearson correlations are calculated between estimated variance and ground truth variance. Top row corresponds to the conventional model, and bottom row to our error model.

In practical applications, not only the quantity of data, but also its quality (in terms of associated uncertainty/variability) is expected to affect GRS identifiability. Applying the model to real experimental data requires an error model that reliably quantifies its data uncertainty. Here, we have considered the sources of data uncertainty, resulting from both biological variability and technical uncertainty (Fig 3A). Based on first principles, we simulated biological variability by varying TF abundances and all the parameters in mechanistic models that describe gene responses (3 *K*_*d*_, *k*_*syn*_, *k*_*proc*_, *k*_*0*_), as well as technical uncertainty by varying sample preparation timepoints and adding noise to the assay measurements (see Methods). Using the set of 26 amplitude perturbations, we simulated two replicate expression patterns for each GRS and captured four time points from each simulation (Fig 3B).

In timecourse data, higher variance is often observed when the timecourse involves sharp increases or decreases. This higher degree of variance may be caused by uncertainty introduced from the time axis (Fig 3C), for example by samples not being collected at the precise timepoints, or a sample of cells responding more slowly due to culture inconsistencies. In principle, the variance of measurements is composed of both a value error and a temporal error, the latter contributing more to the observed variance when there are rapid changes in the timecourse curve than when changes are slow. To account for the contribution of temporal uncertainty, we developed a time-value error model, in which the temporal error is a function of the derivative of the timecourse. As a result, the time-value error model can avoid the under- or over-estimation of error associated with a conventional error model (Fig 3C). A small number of samples do not allow for stable estimation of variance. Here, we combined global trends from all pooled points with an individual point component to achieve a stable point-specific variance estimation. This approach has been widely used when analyzing RNA-seq data (see Methods). For the value uncertainty, we used polynomial regression to capture the value variance-mean relationship. The temporal uncertainty is modeled by a linear Gaussian model (see Methods).

To test the utility of the time-value error model, we applied it to our simulated data and compared results to when the conventional model (which does not consider temporal uncertainty) is applied. By comparing estimated data uncertainty with empirical uncertainty (calculated from 1000 samples), pooling points together can largely improve the estimation accuracy over the directly calculated raw variance, with a Pearson correlation from 0.46 to 0.96 for the time-value model, and from 0.46 to 0.55 for the conventional model (Figs 3D and S4). Interestingly, we clearly see two modes of estimation in the conventional model: one is an over-estimate and the other is an under-estimate, which is consistent with our expectation (Fig 3C and 3D). It also shows that two replicates alone are not able to improve estimation accuracy by combining point-specific information, and more replicates are required to improve the point-specific estimation.

### A workflow to fit GRS models to data

**Fig 4:**
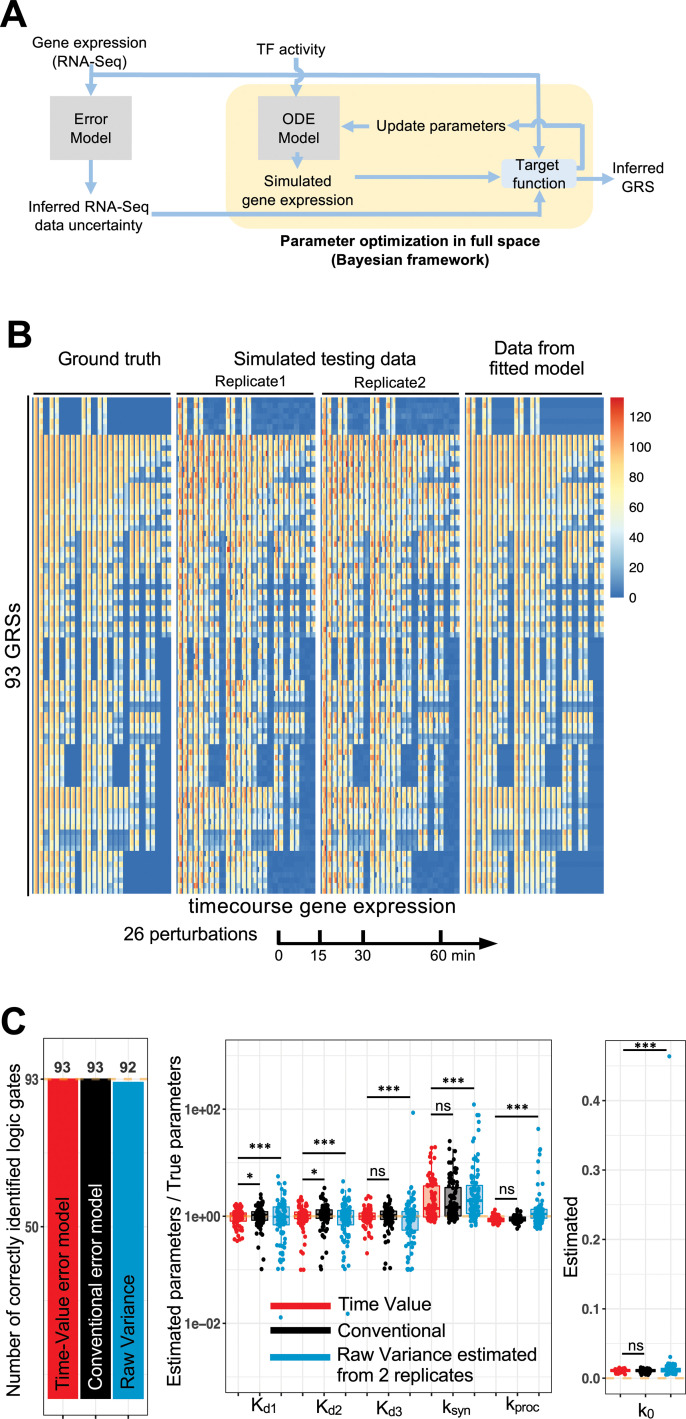
Bayesian framework to parameterize model with data uncertainty. (A) Diagram of the computational pipeline composed of data quality assessment and model parameterization. (B) Gene expression from simulated testing data and fitted model generated data. Rows correspond to the 93 GRSs in the same order as in Fig 2C. Columns corresponds to the 26 amplitude perturbation conditions at 0, 15, 30, 60 min. (C) Bar plot presenting the number of correctly identified logic gate and boxplot of estimated parameters for 93 GRSs fitted models. Percentage of estimated parameters within 2-fold of the true value are for Time-Value model, Conventional model, raw variance respectively: K_d1_ 95%, 91%, 70%; K_d2_ 91%, 84%, 70%; K_d3_ 91%, 90%, 71%. The mean absolute percent deviations are for Time-Value model, Conventional model, raw variance respectively: K_d1_ 24%, 28%, 58%; K_d2_ 26%, 36%, 48%; K_d3_ 23%, 28%, 138%.

Identifying GRSs from experimental data requires not only an error model to characterize data uncertainty, but also an optimization pipeline that enables fitting the model to multiple pairs of input-output datasets of different qualities (Fig 4A). Here, we used a Bayesian framework to derive a likelihood function that serves as the target function and allows fitting the model to datasets of different qualities, while searching the full space for all the parameters (3 *K*_*d*_, *k*_*syn*_, *k*_*proc*_, *k*_*0*_). We applied this optimization workflow to the simulated data, and obtained a fitted model which recapitulates the ground truth data (Fig 4B). Compared to using raw variance, the time-value error model correctly identified all the combinatorial logic gates irrespectively of the associated regulation strength (Fig 4C). Compared to the conventional error model and raw variance, the time-value error model better constrained the estimated regulation strengths within two-fold of the true values (Fig 4C). Further, we found that this improved performance of the time-value error model in GRS parameter estimation over the conventional model and raw variance is irrespective of the degree of uncertainty or noise level (S5A Fig). Interestingly, we found that when we consider both combinatorial logic gate and regulation strength, a total of 15 GRSs are mis-classified when using either the conventional model or the raw variance. These 15 GRSs are associated with clusters whose GRSs we previously found are hard to distinguish in our theoretical analysis (S5B Fig), like triple AND gate, and single TF regulated clusters. In addition, the confused GRSs are mis-classified mostly with their neighbors characterized by the tree structure (S5B Fig).

### Guidelines for designing stimulation/perturbation studies

**Fig 5:**
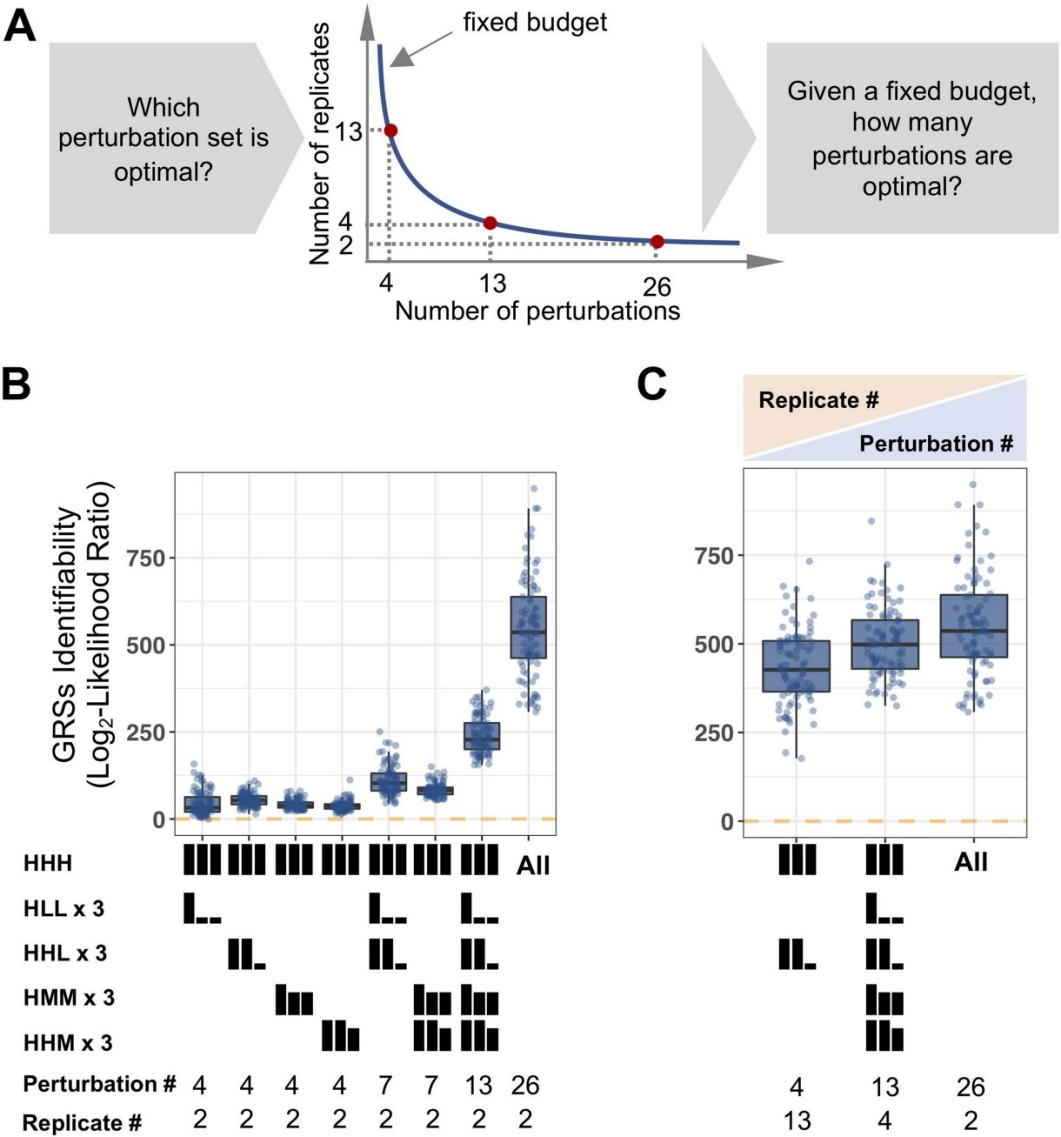
Principles of designing the experimental perturbation studies. (A) Schematic of the relationship between the number of replicates and perturbation conditions given a fixed budget to generate for example 52 datasets. (B) Effect of combining different perturbation sets on the identifiability (defined as the log2 likelihood ratio between the ground truth and the most similar alternative GRS) of the 93 GRSs. Testing 2 replicates for each condition, the number of perturbation sets determines GRS identifiability. Mathematically, we defined identifiability of each GRS as the lowest squared Euclidian distance between the gene expression responses of the ground truth GRS and any other GRS. (C) Comparison of the trade-off between the number of replicates and number of perturbation conditions for the identifiability of the 93 GRSs. When 52 datasets can be generated, employing more perturbation datasets is preferable to having more replicates for fewer perturbation sets.

To identify GRSs from experimental data, not only the quality but also the quantity of available data and the type of perturbation conditions matter. Specifically, we showed that GRSs can produce very different gene expression patterns under different perturbation conditions (Figs 1F and 2B), and thereby leads to different identifiability under those conditions. Therefore, we wondered whether we could determine which sets of perturbation conditions are most informative to distinguish GRSs (Fig 5A). In practice, researchers, given a fixed budget to generate for example 52 datasets, need to decide whether to generate a large number of replicates to reduce experimental error prioritizing more perturbations that would provide additional perturbation-response information (Fig 5A).

Examining the identifiability (defined as the log_2_ likelihood ratio between the ground truth and the most similar alternative GRS) using simulated, imperfect data (due to biological variability and measurement uncertainty) from all possible perturbation combinations, we first compared GRS identifiability for different sets of single or double knockout or knockdown conditions (HLL, HHL, HMM, HHM), which correspond to setting TF perturbation or activity levels to fully activated (H) as in wild-type, low (L) for knockouts, or medium (M) for knockdowns. We found that the single knockout (HHL) is the most informative of these perturbations (Fig 5B), but combining the three single and three double knockout conditions (HLL, HHL) provides a substantial boost to distinguishing the 93 GRSs, more so than when knockdowns (HHM, HMM) alone were used. However, combining knockdowns (HHM, HMM) can help distinguish the weakly identifiable GRSs by distinguishing the regulation strength features, with the comprehensive set of 26 perturbations (including mixed knockout and knockdown) providing a further boost in identifiability.

Next, we considered how to optimally allocate resources that would allow for 52 datasets. If only 4 perturbation conditions (of WT and three single knockout conditions) are employed, providing them in 13 replicates substantially increases identifiability (median GRS identifiability metric of 427 vs. 54). However, spreading resources to more perturbations (either 13 × quadruplets, or better 26 duplicates), will further improve identifiability (median GRS identifiability metric 498, 536 vs. 427) (Fig 5C). Further, we found generating more perturbations is still more informative even with the small number of datasets (S6 Fig). These studies suggest that simulations may be helpful in prioritizing limited resources for designing experimental studies to elucidate GRSs based on input-output data.

### GRSs of immune response genes

We applied the newly developed computational workflow to identify GRSs for immune response genes in bone marrow derived macrophages (BMDMs). NFκB, IRF, and MAPK are three signaling pathways governing immune response gene expression. We obtained nascent RNA expression from chromatin associated RNA-seq (caRNA-seq) by processing recently deposited data [19]. We measured NFκB and IRF3 activities, and inferred MAPK-regulated transcription factor activity (given the absence of a direct TF assay) from the expression patterns of well-established immediate early response genes (Egr1, Fos, and Dusp4) that are neither NFκB, nor IRF targets [18,19] (S7A Fig). Here, we focused on the primary immune response genes (see Methods for selecting induced genes) within the first hour after stimulation, to avoid potential secondary effects from signaling cascades or feedbacks.

**Fig 6:**
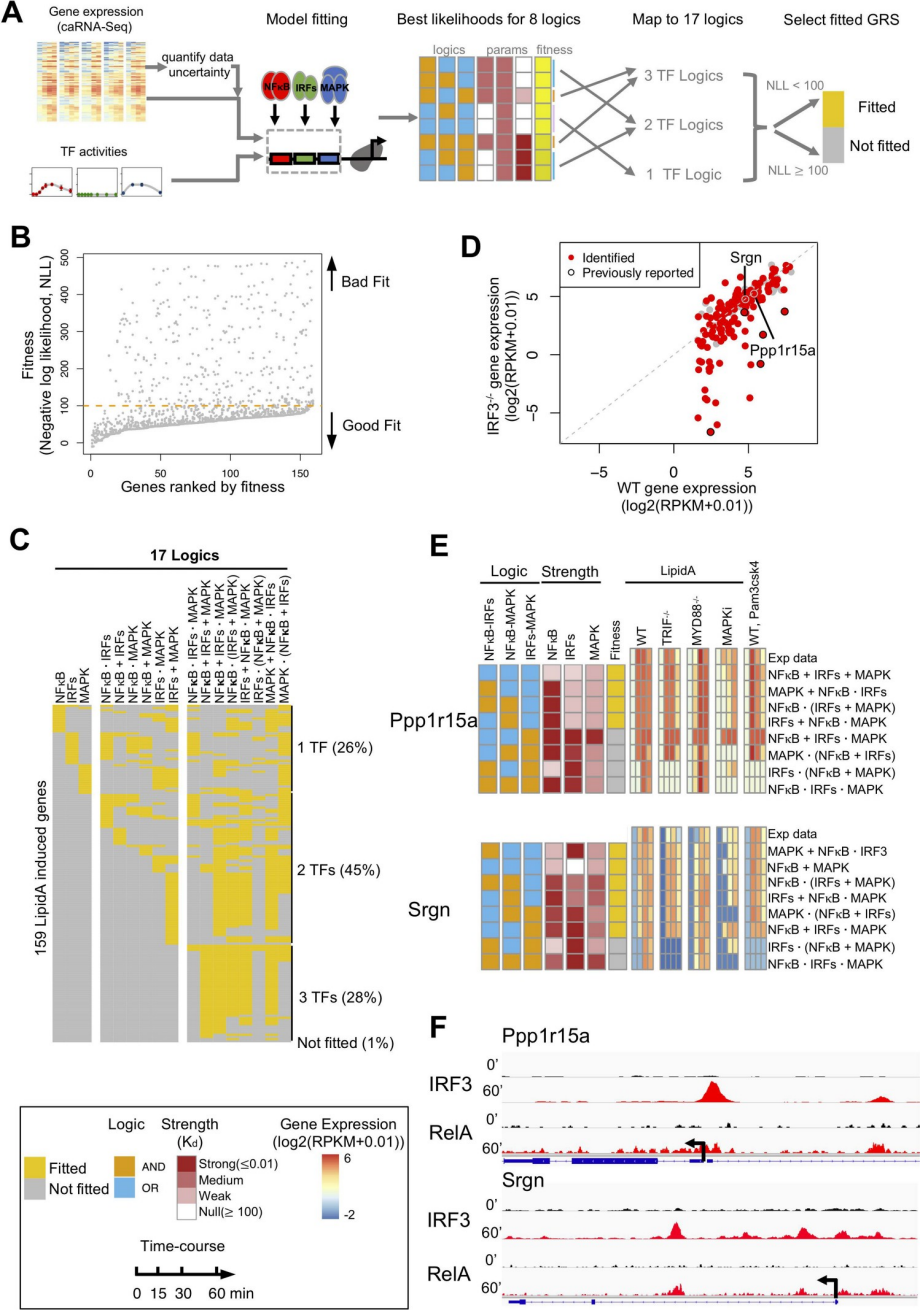
Mapping GRSs involving NFκB, IRFs, MAPK to endotoxin-induced immune genes. (A) Scheme of fitting models to experimental data. Gene expression data and TF activities for different stimuli were used to fit 8 possible model topologies and best likelihoods are determined; this was followed by mapping to 17 possible logics (Fig 1D), which were then determined to whether they fit the data, or not. (B) The plot of fitness (negative log likelihood) of 8 triple TF logic gates for all lipid A-induced genes identified by [19]. An arbitrary threshold to select fitted logic gates is marked as an orange dashed line. (C) The plot of fitted GRS logic gates for lipid A-induced genes identified by [19]. Only 26% of these genes fit a GRS governed by a single TF; most require GRS models that are controlled by at least two. (D) Peak expression of IRF3^-/-^ is assessed against WT peak expression for all the lipid A-induced genes. Genes that are identified as potentially synergistically regulated by NFκB and IRFs are marked in red. The 5 previously reported IRF3-NFκB-regulated genes [19] are marked with a black circle. (E) For two example genes, heatmaps of experimentally measured caRNA-seq time-course data and simulation data by the best-fit GRS models, ordered by fit quality from top (best) to bottom. (F) IGV genome browser tracks of IRF3 and RelA ChIP-seq data in resting and 60 min lipid A-stimulated macrophages of the two genes.

We first quantified data uncertainty of the time-course caRNA-seq data with the newly developed error model (Fig 3), and then estimated parameters from models with 8 logics with the described Bayesian framework (Fig 4). We mapped the 8 triple logics back to the original 17 logics (Fig 1D) by identifying dual or single TF logics *via* regulation strengths (Fig 6A). Given the available data, most genes were matched to multiple GRSs (Figs 6B and S7B). However, interestingly, our analysis suggested that only 26% of genes were matched to GRSs governed by a single TF, whereas 74% of genes could only be accounted for by logics involving 2 or 3 TFs (Fig 6C). This suggests that combinatorial gene regulation is much more common than previously reported [18,19].

Next, we focused on genes that are potentially synergistically regulated by NFκB and IRFs, i.e. genes to which any of the 5 logics may apply: NFκB AND IRFs, NFκB AND IRFs AND MAPK, NFκB AND (IRFs OR MAPK), IRFs AND (NFκB OR MAPK), MAPK OR NFκB AND IRFs. We identified not only all 5 previously reported NFκB-regulated genes that are IRF-dependent (Fig 6D) [19], but also a bigger set of additional genes. Many of these showed potential involvement of MAPK that may compensate for loss of IRFs, and thus show little gene reduction in IRF3^-/-^ cells compared to wild type (Fig 6D). This illustrates the limitation of using knockout data (IRF3^-/-^) as a defining feature of whether a gene is regulated the TF, or, in this case, IRF-NFκB AND gate genes. Ppp1r15a and Srgn are examples of such genes that we identified as being potentially synergistically regulated by NFκB and IRFs with little reduction in expression in IRF3^-/-^ cells (Figs 6D and 6E and S7C). Further analysis of ChIP-Seq data revealed induced binding of both RelA and IRF3 in the promoter and gene body of both genes (Fig 6F), confirming the potential regulation by both RelA and IRF3. To elucidate the regulation of these genes more precisely and identify unique GRS models requires additional perturbations, such as combinatorial knockout or inhibition of MAPK and IRF3.

## Discussion

Over the past two decades a wealth of cellular transcriptomic and epigenomic data have been generated but there has only been limited progress in understanding the multi-TF gene regulation networks (GRNs) of mammalian cells [1,25]. Here we have presented a theoretical analysis and a computational framework to examine the feasibility to infer abstracted mechanistic models of GRSs as stepping stones to develop the more detailed mechanistic understanding of GRNs. We show that GRS model identifiability is a function of the quantity and quality of data, and present a GRS model fitting computational workflow that includes an improved error model. We then apply this workflow to experimental data of innate immune gene expression in macrophages, and demonstrate its utility by revealing that the data supports alternative hypotheses that were previously missed by conventional analysis approaches. This work has been inspired by multiple previous studies to obtain mechanistic insights from genome wide genomic data using mathematical models involving logic gates [6]. Our work in particular examined the identifiability of different mechanistic models by measuring TF activity directly, and by considering different data uncertainty levels. Therefore, our work serves as a stepping stone to gain finer mechanistic insights of gene regulation.

Our computational analysis revealed a non-intuitive mapping relationship between model/mechanism and gene expression responses. We showed that not all GRSs are equally identifiable, and nor are all GRS regulatory features equally identifiable. For example, GRSs formed by differential regulatory strengths are much harder to distinguish within the triple AND gate than within OR gates. But the triple AND gate logic feature is readily identifiable from all other GRSs with less available data. By displaying the model/mechanism-gene expression mapping relationship as a hierarchical tree, we found core regulation motifs that are hallmarks of specific GRS clusters and therefore play dominant roles in determining the input-output relationship. This finding suggested that some important insights can be gained reliably even when available datasets do not allow for the identification of a single GRS. For example, the triple AND gate and the double AND gate as core regulation motifs can be easily identified even when distinct GRSs that differ in the regulation strengths within each motif cannot be determined easily. Similarly, the emergent dynamical systems properties (e.g. perfect adaptation) may be mediated by diverse networks that nevertheless share common network motifs, as revealed by a function-topology map [26]. It is interesting that in our mechanism-gene expression map, we similarly found conserved core regulation motifs enriched in GRSs with similar input-output relationships. As such core regulation motifs determine their function, they are readily identifiable even from limited datasets.

In considering input-output datasets that involve perturbations at a given timepoint, we found it useful to precisely decompose value and temporal uncertainty for timecourse data. Our analysis of the “time-value” error model with synthetic data indicates that accurately accounting for data uncertainty can improve model identifiability by correctly penalizing less reliable data, but avoiding under- and over-estimates of the error associated with value. The time-value error model may be useful for timecourse data involving sharp changes, observed in data sets from highly dynamic biological processes like signal transduction or gene activation in response to stresses, immune threats or growth factors, where temporal error can greatly contribute to the data uncertainty.

After developing a practical computational workflow, we used simulated datasets to provide some guiding principles for experimental design for generating the most informative stimulus-response datasets. Model-guided experimental design is addressed in a rich literature [27–29], and some of our insights are intuitive. First, it is clear that given a limited budget, a greater number of distinct perturbation conditions should be prioritized over more replicates that would diminish error uncertainty. As an error model can be parameterized from replicates of a single perturbation condition, duplicates of other conditions primarily serve the purpose of ensuring the experiment did not in some way fail. Second, although perturbations that involve complete knockout may better distinguish GRSs in general, perturbations with partial activities are particularly helpful for determining regulation strengths. These involve dose response studies or siRNA-mediated knockdown rather than complete knockout. Not surprisingly, we found that the optimal experimental perturbation condition set depends to some extent on the GRSs to be identified. This suggests that the optimal strategy is an iterative approach. To begin with, a set of perturbations that activate TFs singly or doubly can first segregate GRSs into multiple clusters. In the following round, based on these segregated clusters of GRSs and their shared features, additional perturbations may be selected to further dissect these GRSs and find other associated regulatory features.

Our work also provides guiding principles for mapping other types of observed data to the molecular network. The general approach outlined here—generating a model-gene expression map by comparing all possible hypotheses and fitting the model to integrated data sets—can be extended beyond the question of TF combinatorial regulation of transcription initiation. For example, epigenomic datasets that describe chromatin remodeling (ATAC-Seq), TF-DNA interactions (ChIP-Seq), chromatin conformation (Hi-ChIP, PLAC-seq), and protein-RNA interactions (CLIP-Seq), may be used to distinguish alternate models of multiple or sequential steps of gene regulation. Furthermore, the abstracted combinatorial models described here may be iteratively refined for example by addressing the TFs regulating different and sequential kinetic steps leading to transcriptional initiation [30,31], and may be used to identify additional regulatory mechanisms.

Applying the computational workflow to publicly available and newly generated input-output data of macrophages responding to immune stimuli illustrates the power of this quantitatively rigorous analysis workflow. Previous purely experimental studies relied heavily on knockout data to infer the involvement of a transcription factor. However, biological systems are characterized by overlapping functions of its components, a feature that confers functional robustness [32]. Hence, the regulatory role of a transcription factor on a particular gene is not reliably reflected in its functional requirement. In the case of pathogen-response gene expression, the potential for functional overlap manifests itself in many more genes being potentially combinatorially regulated than previously reported [18,19]. Indeed, physical binding data of the transcription factor IRF3 supports the notion that this transcription factor is more widely contributing to gene regulation than previously reported [19,33]. Further, the described computational workflow is extensible for keeping rigorous account of which GRSs may be excluded for each immune response gene as additional datasets become available.

In sum, as we lack a quantitative understanding of the molecular mechanisms governing many important biological regulatory systems, which would enable detailed regulatory network models, we describe here an approach for distinguishing quantitatively between alternate regulatory strategies using input-output datasets. Such an approach connects biological knowledge to data-driven approaches, and may prompt and guide subsequent mechanistic experimental and mathematical modeling studies. Considering TF combinatorial regulation alone, there are numerous examples that may benefit from the described workflow. In macrophages, the response to pathogens and cytokines is mediated by a handful of inducible TFs that are thought to engage in AND and OR gates [19,20]. Similarly, in hemogenic reprogramming or in induced pluripotent stem cell reprogramming initiated by three Yamanaka factors, combinatorial TF regulation triggers a cascade of successive molecular events that mediate the biological process [34,35]. Thus, the described computational framework may serve as a manual to gain quantitative understanding of gene regulation, and provide guidance for optimal experimental designs.

## Methods

### Model formulation

In this work, we quantitatively studied combinatorial GRSs formed by three TFs. Hence, we modeled nascent RNA expression from activated gene by considering RNA synthesis and first order RNA processing. RNA abundance dynamics can be modeled using a single ODE:

$$\frac{dRNA}{dt}=k_{syn}∙f(t)-k_{proc}∙RNA$$ (1)

Here *k*_*proc*_ represents the nascent RNA processing rate, *k*_*syn*_ a constant synthesis rate that is modulated by the fractional promoter activity *f(t)*, which includes both combinatorial regulation from the GRS and some basal activity_:_

$$f(t)=(1-k_{0})∙GRS(TFs(t))+k_{0}$$ (2)

*f(t)* describes how TFs regulate promoter activity, which is composed by a logic gate function GRS that depends on the TFs activities and some basal promoter activity denoted *k*_*0*_.

### Formulation of logic gates

We considered all possible synergistic (AND) and non-synergistic (OR) gene regulation strategies involving three TFs. As it has been shown in Fig 1D, there are 3 single logics (one for each TF), and 6 dual logics by considering two options “∙” and “+” two options for the 3 pairs of the TFs (2 ∙ 3 = 6). The triple logics come from the 6 dual logics with an additional “∙” and “+” for the third and by removing 4 redundant logics (2 of TF1+TF2+TF3, and 2 of TF1∙TF2∙TF3), leading to the final 8 triple logics (2 ∙ 6–4 = 8). The single and dual logics can be represented by a triple logic with null regulation strength of one or two TFs, see Fig 1D. This can also be derived by Boolean algebra, for example, TF1+TF2+TF3 = TF1+TF2 with TF3 = 0. Therefore, considering the 8 triple TFs logics is enough to represent all single, dual and triple logics. As synergistic (AND) and non-synergistic (OR) gene regulations can be modeled with thermodynamic models [15,17,18], the eight the derived logic gates represents four main structures that can be modeled by these formulas:

$$TF_{1}\;AND\;TF_{2}\;AND\;TF_{3}:\;G=\frac{\left[{TF}_{1}(t)\right]}{K_{d1}+[{TF}_{1}(t)]}∙\frac{\left[{TF}_{2}(t)\right]}{K_{d2}+[{TF}_{2}(t)]}∙\frac{\left[{TF}_{3}(t)\right]}{K_{d3}+[{TF}_{3}(t)]}$$ (3)

$$TF_{1}\;AND\;(TF_{2}\;OR\;TF_{3}):\;G=\frac{\left[{TF}_{1}(t)\right]}{K_{d1}+[{TF}_{1}(t)]}∙\left(1-\frac{K_{d2}}{K_{d2}+[{TF}_{2}(t)]}∙\frac{K_{d3}}{K_{d3}+[{TF}_{3}(t)]}\right)$$ (4)

and similarly by rotation for TF_2_ AND (TF_1_ OR TF_3_) and for TF_3_ AND (TF_1_ OR TF_2_):

$$TF_{2}\;AND\;(TF_{1}\;OR\;TF_{3}):\;G=\frac{\left[{TF}_{2}(t)\right]}{K_{d2}+[{TF}_{2}(t)]}∙\left(1-\frac{K_{d1}}{K_{d1}+[{TF}_{1}(t)]}∙\frac{K_{d3}}{K_{d3}+[{TF}_{3}(t)]}\right)$$ (5)

$$TF_{3}\;AND\;(TF_{1}\;OR\;TF_{2}):\;G=\frac{\left[{TF}_{3}(t)\right]}{K_{d3}+[{TF}_{3}(t)]}∙\left(1-\frac{K_{d1}}{K_{d1}+[{TF}_{1}(t)]}∙\frac{K_{d2}}{K_{d2}+[{TF}_{2}(t)]}\right)$$ (6)

$$TF_{1}\;OR\;(TF_{2}\;AND\;TF_{3}):\;G=1-\frac{K_{d1}}{K_{d1}+[{TF}_{1}(t)]}∙\left(1-\frac{\left[{TF}_{2}(t)\right]}{K_{d2}+[{TF}_{2}(t)]}∙\frac{\left[{TF}_{3}(t)\right]}{K_{d3}+[{TF}_{3}(t)]}\right)$$ (7)

and similarly by rotation for TF_2_ OR (TF_1_ AND TF_3_) and for TF_3_ OR (TF_1_ AND TF_2_):

$$TF_{2}\;OR\;(TF_{1}\;AND\;TF_{3}):\;G=1-\frac{K_{d2}}{K_{d2}+[{TF}_{2}(t)]}∙\left(1-\frac{\left[{TF}_{1}(t)\right]}{K_{d1}+[{TF}_{1}(t)]}∙\frac{\left[{TF}_{3}(t)\right]}{K_{d3}+[{TF}_{3}(t)]}\right)$$ (8)

$$TF_{3}\;OR\;(TF_{1}\;AND\;TF_{2}):\;G=1-\frac{K_{d3}}{K_{d3}+[{TF}_{3}(t)]}∙\left(1-\frac{\left[{TF}_{1}(t)\right]}{K_{d1}+[{TF}_{1}(t)]}∙\frac{\left[{TF}_{2}(t)\right]}{K_{d2}+[{TF}_{2}(t)]}\right)$$ (9)

$$TF_{1}\;OR\;TF_{2}\;OR\;TF_{3}:\;G=1-\frac{K_{d1}}{K_{d1}+[{TF}_{1}(t)]}∙\frac{K_{d2}}{K_{d2}+[{TF}_{2}(t)]}∙\frac{K_{d3}}{K_{d3}+[{TF}_{3}(t)]}$$ (10)

#### Enumeration of representative GRSs

Our focus here is the synergistic and/or non-synergistic combinatorial regulation by 3 TFs to produce a specific GRS. Hence for each regulatory logic, we considered that each TF may have one of three regulation strengths (strong (S), medium (M), and weak (W)). Thus, we considered a total of 27 regulation strength combinations for 3 TFs and 8 logic gates, yielding a total 216 possible GRSs. Of these 216 GRSs, several may in fact not be efficiently activated. For example, AND gate configurations in which one component has a “Weak” regulatory strength cannot be activated well within the present scheme. In this way, we identified 69 poorly activated GRSs and removed them from further consideration. In addition, several GRSs were found to be logically equivalent. For example, the TF1 is logically equivalent to either a triple OR gate in which TF2 and TF3 have “Weak” regulatory strength, or a TF1 or (TF2 and TF3) gate in which TF2 or TF3 have “Weak” regulatory strength. In this way we removed 54 redundant GRSs. These first principle considerations led us to a list of 93 potentially identifiable GRSs (see S2 Fig).

### Perturbations

We defined perturbations as following functions.


$$Simple\;perturbation:\;f(t)=\left\{ \begin{array}{c} 1,high\\ 0,low \end{array}\right.$$ (11)

In addition to the simple perturbation, we have:

Amplitude:f(t)=0.5,0≤t≤60 (12)

$$Gradient:\;f(t)=\frac{t}{60},0\leq t\leq 60$$ (13)

$$Delayed:\;f(t)=\left\{ \begin{array}{c} 0,0<t\leq 30\\ 1,30<t\leq 60 \end{array}\right.$$ (14)

$$Transient:\;f(t)=\left\{ \begin{array}{c} 1,0<t\leq 30\\ 0,30<t\leq 60 \end{array}\right.$$ (15)

### GRS simulation

In our work, we defined GRS as the combination of logic gate and regulation strength parameters, which together determine gene expression levels. For testing purpose, we assume TFs activities range from 0 to 1 (i.e. TFs are normalized to avoid assay specific dynamic span) and define the high level as 1, the medium level as 0.5, and the low level as 0. Based on Hill function, we define regulation strength ranging from weak to strong with strong corresponding to *K*_*d*_ = 0.1, medium to *K*_*d*_ = 1, and weak to *K*_*d*_ = 10, given that *K*_*d*_ has minimal effect on promoter activity outside this range. We consider gene with a short RNA processing rate (7min, i.e. *k*_*proc*_ = 0.1 min^−1^). Here, we simulate a timecourse gene expression at 0, 15, 30, 60 min, but our approach can be generalized into any time points served for the interests of experiments. To compare all the GRSs, we simulated resulting gene expression with *k*_*syn*_ = 1 RPKM/min (where RPKM means reads per kilobase per million of mapped reads), and normalized the gene expression such that its maximal value corresponds to 100 across all the perturbation conditions. This is equivalent to adjusting *k*_*syn*._ In Table 1, you can find a summary of the different parameters used for the simulations:

**Table 1:** Model Parameters.

Parameters	Description	Range	Value
[TFs] (Inputs)	TF amplitude	High Medium Low	1 0.5 0
*K*_*d*_	TF regulation strength	Strong Medium Weak	0.1 1 10
*k*_*0*_	Basal gate value		0
*k*_*syn*_	Nascent mRNA synthesis rate		1 RPKM/min
*k*_*proc*_	Nascent mRNA processing rate		0.1 min^-1^

### Computational analysis

We define the distance between two gene expression profiles as the squared Euclidean distance for each timepoints across all perturbation conditions:

$$d({gene}_{i},{gene}_{j})=\sum_{p\in perturbations}\sum_{t\in timepoints}{\left({ge}_{i,p}(t)-{ge}_{j,p}(t)\right)}^{2}$$ (16)

where *ge*_*i*,*p*_(*t*) means gene expression of gene *i* for perturbation *p* at time *t*. We used a hierarchical clustering with single linkage approach to cluster GRSs based on their gene expression profile. With single linkage approach, the distance between two clusters of GRSs is defined as the minimum distance of inter-pairs, as we considered two groups of GRSs is not identifiable if one inter-pair distance is below a certain tree height threshold, called in the rest of the manuscript separation threshold.

### GRS analysis with randomly sampled parameters

We followed the same procedures described in GRS simulation to examine GRS with randomly sampled parameters. Specifically, we generated 1000 sets of parameters of each logic gate by sampling uniformly from parameter space (described in Table 2) in logarithm scale. We only selected the activatable GRS for the downstream analysis. The activatable GRS are defined as GRS that can produce an output higher than the maximum value of previously identified 69 poorly activated GRS (here the threshold is 1.66 for GRS output) when the 3 input TFs are fully active (i.e. activity of 1). As sampling effects of *k*_*syn*_ will be counteracted by the normalization of gene expression, the random sampling of *k*_*syn*_ was replaced by constant number (1 RPKM/min).

**Table 2:** Parameter ranges.

Parameters	Description	Sampling Range
*K*_*d*_	TF regulation strength	0.01–100
*k*_*0*_	Basal gate value	0–1
*k*_*proc*_	Nascent mRNA processing rate	0.01 *min*^−1^–10 *min*^−1^

### Error model

#### Reliable point specific estimation

To achieve reliable point specific estimation from small sample size, we leveraged global trend from all the points to stabilize the estimation. For this purpose, we used Bayes’ rule to combine global trend (prior distribution) with point specific information using maximum likelihood estimation (MLE) to generate a stabilized estimate (posterior distribution). This empirical Bayes approach have been broadly used in handling small sample problem in bulk and single cell RNA-seq data, including by DESeq2 [36], voom [37], and BASiCS [38].

Specifically, we first estimate the error parameters ***ξ***_***prior***_, which are defined based on the error model described later, from global trend by pooling all the points (i.e. all genes and time points) together. Next, we combine the global trend with data of each time point *i* to estimate the posterior distribution ***ξ***_***i*,*posterior***_ of each time point, which is given by:

$$\xi_{i,posterior}=\underset{\xi_{i}}{\mathrm{argmax}}\;L(\xi_{i})P(\xi_{i})$$ (17)

Where ℒ(***ξ***_***i***_) is the likelihood function that represents information from each time point *i*, and *P*(***ξ***_***i***_) corresponds to the prior knowledge on the distribution of ***ξ***_***i***_ which we assume to be normal with mean ${\hat{\xi }}_{prior}$ estimated from global trend, and $\sigma_{\xi_{prior}}^{2}$ as variance. Here, $\sigma_{\xi_{prior}}^{2}$ allows scaling the importance of the global trend over the point specific information. We empirically chose the prior variance based on our confidence of how point-specific information contributes to the estimation. Specifically, we first start with a very small prior variance 0.00001 so that the posterior would almost be the same. Then we gradually increase the prior variance, thereby putting more weight on point-specific information, and examine whether the performance (correlation between ground truth and estimation) is improving. By doing so, we may choose the optimal prior variance given the number of available replicates. If there are few replicates (less than 10), then we tend to put high weight i.e. confidence (small $\sigma_{\xi_{prior}}^{2}$) on the global trend so that the final posterior estimation is similar to the global trend. If a large number of replicates are available (more than 10), we may increase the weight of the point specific information (larger $\sigma_{\xi_{prior}}^{2}$).

#### Uncertainty estimation

Uncertainty originates from various sources. Here, we have considered uncertainties coming from biological variabilities including TF abundance variations, gene response time variations, chemical parameters variations, and technical uncertainty including sample preparation timepoints variation and assay measurement variation. We separately modeled them into value uncertainty (TF abundance variations, chemical parameters variations, and assay measurement variation), and temporal uncertainty (gene response time variations and sample preparation timepoints variation), as those two sources of uncertainties are orthogonal (value and temporal), and have two different mathematical forms (temporal uncertainty is curve shape dependent, and value uncertainty is not).

#### Temporal uncertainty estimation

To model the temporal uncertainty of a point (*t*, *y*_*t*_) due to potential uncertainty in the exact time the measurement was done, we assume that it follows a Gaussian distribution with mean t and standard deviation *σ*_*t*_. The resulting uncertainty in y due to the uncertainty in t is denoted by *σ*_*temporal*_.

For a small neighborhood around *t*, the curve, which passes the point in such a small region, can be approximated by a line (linearization) with the slope as tangent of the curve. Therefore with the assumption that the uncertainty of *t* follows a Gaussian distribution, then y will also follow a Gaussian distribution, where variance is given by [39]:

$${\sigma_{temporal}}^{2}={{Slope}^{2}{∙\sigma }_{t}}^{2}$$ (18)

This can be considered as an error propagation function, which is:

$${\left(\Delta y\right)}^{2}={\left(Jacobian\cdot \Delta t\right)}^{2}$$ (19)

#### Value uncertainty estimation

As we know multiple uncertainty sources can cause observed value uncertainty, we assume the overall observed value uncertainty follows a Gaussian distribution.

To obtain robust value uncertainty estimation, we modeled mean-variance relationship by a polynomial regression, where $\sigma_{value}^{2}$ is representing value variance that can be predicted by the mean of point *μ*. This helps stabilize the estimation of variance by leveraging its dependency relationship with robust estimator, mean. Similar approach to capture mean-variance relationship was introduced by DESeq2 [36], where it uses fixed relationship, and BASiCS [38], where it uses more flexible semi-parametric approach with Gaussian radial basis function (GRBF) kernels. We use polynomial regression as it can capture the constant, linear, and quadratic variance components. It is given by:

$$\sigma_{value}^{2}=f(\mu )=\alpha_{0}+\alpha_{1}∙\mu +\alpha_{2}∙\mu^{2}$$ (20)

Here, we only include the first two orders, as this already captures well the mean-value variance dependency. Higher order terms can be added when dealing with more complex datasets.

#### Distribution of gene expression

We separately modeled background and induced gene expression distribution. These two parts are usually caused by different sources, and exhibit different level of uncertainty. In addition, the distribution for background uncertainty is desired to have ℝ_≥0_ as its support, as all measurements are positive. Therefore, the distribution of gene expression is modeled by gamma and normal distributions, depending on the expression level, which is given below:

$${gene}_{i}∼\left\{ \begin{array}{c} \Gamma (k_{bg,i},\theta_{bg,i}),if\;\mu_{i}\leq \eta \\ N(\mu_{i},\sigma_{induc,i}^{2}),if\;\mu_{i}>\eta \end{array}\right.$$ (21)

Here, we connect data mean and variance to the gamma distribution’s mean and variance to parameterize *k*, *θ*:

$$(k_{bg,i}-1)∙\theta_{bg,i}=\mu_{i}$$

$$k_{bg,i}∙{\theta_{bg,i}}^{2}=\sigma_{bg}^{2}$$ (22)

where,

$${\sigma_{induc,i}^{2}=\alpha }_{induc,0,i}+\alpha_{induc,1,i}∙\mu_{i}+\alpha_{induc,2,i}∙{\mu_{i}}^{2}+{Slope}_{i}^{2}∙\sigma_{induc,t,i}^{2}$$ (23)

In addition, we set the threshold *η* to be 3 RPKM.

### Implementation of error model

#### Simulation of data uncertainty

We followed the first principle to generate data uncertainty from both biological variability and technical uncertainty. We generated biological variability by varying TF amplitude, gene response time and parameters of the model by sampling from the distribution specified in Table 3. We generated technical uncertainty by varying sample preparation timepoints, and adding noise to the assay measurement, as specified in Table 3.

**Table 3:** Simulated uncertainty.

Uncertainty Source	Form	Distribution
TF amplitude	*TF*∙(1+*ε*)	ε∼N(0,0.0004)
Gene response time	*t*+*ε*	ε∼N(0,25)
Parameters (*K*_*d*_, *k*_*syn*_, *k*_*proc*_)	*Parameters*∙*e*^*ε*^	ε∼N(0,0.0004)
Parameters (*k*_0_)	*k*_0_+*ε*	*ε*~*U*(0,0.02)
Sample preparing timepoints	*t*+*ε*	ε∼N(0,25)
Assay measurement	*y*+*ε*	$\varepsilon ∼N(0,0.01∙y^{2})$

#### Slope estimation

To reliably estimate the slope of the curve, we first interpolated the curve with a piecewise cubic hermite function (pchip function in the R signal package), and then estimated the slope from the interpolated curve. This allowed us to leverage information from all points and the shape of the curve. Specifically, the slope is calculated by the central difference:

$$Slope=\frac{\Delta y_{fit}}{\Delta t}=\frac{y_{fit,t+\frac{\Delta t}{2}}-y_{fit,t-\frac{\Delta t}{2}}}{\left(t+\frac{\Delta t}{2})-(t-\frac{\Delta t}{2}\right)}$$ (24)

The choice of Δ*t* depends on temporal uncertainty level, as one can imagine we should take bigger Δ*t* when temporal uncertainty is high. Here, we set $\Delta t=2∙{\hat{\sigma }}_{t}$ (${\hat{\sigma }}_{t}$ is the unbiased estimated temporal uncertainty). We set the slope to be 0 for sample at time 0, as it comes from unstimulated cells and should correspond to steady state. We interpolate the time series to range from 0 to 80 min to allow for a better estimate of the slope for the last time point.

### Error inference

#### Likelihood function

We first derived the likelihood function for the inference with parameters $\xi =(\sigma_{bg}^{2},\alpha_{0},\alpha_{1},\alpha_{2},\sigma_{t}^{2})$. The probability distribution for measured ***y***_***i***_ at time point *i* is given by:

$$y_{i}{|\xi }_{i},{Slope}_{i}^{2},\mu_{i}∼\left\{ \begin{array}{c} \Gamma (y_{i}|\frac{{\mu_{i}}^{2}}{\sigma_{i,bg}^{2}},\frac{\sigma_{i,bg}^{2}}{\mu_{i}}),if\;\mu_{i}\leq 3\\ N(y_{i}|\mu_{i},\sigma_{total,i}^{2}),if\;\mu_{i}>3 \end{array}\right.$$ (25)

Where $\mu_{i}={\bar{y}}_{i}$. Therefore, the point likelihood ℒ_*point*_ is given by:

$$L_{point}(\xi_{i})=I(\mu_{i}\leq 3)\Gamma \left(y_{i}|\frac{{\mu_{i}}^{2}}{\sigma_{i,bg}^{2}},\frac{\sigma_{i,bg}^{2}}{\mu_{i}}\right)+I(\mu_{i}>3)N(y_{i}|\mu_{i},\sigma_{total,i}^{2})$$ (26)

Here, Γ and N are used to denote the probability density function (PDF) of gamma and gaussian distributions respectively. We will use this notation to describe both the distribution or its PDF based on the context in the rest of the manuscript.

Next, we estimated ***ξ***_*prior*_ from global trend of points using global likelihood ℒ_*global*_, which is given by the multiplication of the point likelihood from all the perturbations *p*, genes *g* and time points *t*. ℒ_*global*_ is given by:

$$L_{global}(\xi_{prior})=\prod_{p}\prod_{g}\prod_{t}L_{point}(\xi_{prior})$$ (27)

Finally, we use Bayes’ rule to estimate ***ξ***_*i*,*posterior*_ by combining global trend with point specific information. We assume ***ξ***_*prior*_ follows a normal distribution, with mean as ${\hat{\xi }}_{prior}$ and variance as $\sigma_{\xi_{prior}}^{2}$. Based on the confidence of prior information, we can adjust $\sigma_{\xi_{prior}}^{2}$ to change the weights of point information and global trend. It is given by:

$$\xi_{i,posterior}=\underset{\xi_{i}}{\mathrm{argmax}}\;L(\xi_{i})P(\xi_{i})=\underset{\xi_{i}}{\mathrm{argmax}}\;P(y_{i}|\xi_{i},{Slope}_{i}^{2},\mu_{i})N(\xi_{i}|{\hat{\xi }}_{prior},\sigma_{\xi_{prior}}^{2})$$ (28)

For the computational efficiency during optimization, we convert all the target function as negative log, and use Limited-memory BFGS (L-BFGS) method which is implemented in the optim function in R for optimization. More details can be found in the parameter estimation and model selection part.

As MLE of variance is a biased estimator, we have corrected it to the unbiased estimator by:

$${\hat{\sigma }}_{total}^{2}=\frac{N}{N-1}{\hat{\sigma }}_{total,MLE}^{2}$$ (29)

where *N* is the number of replicates (here N = 2). All the estimated parameters for variance have been corrected by:

$$\hat{\xi }=\frac{N}{N-1}{\hat{\xi }}_{MLE}\;\mathrm{with}\;\hat{\xi }=({\hat{\sigma }}_{bg,MLE}^{2},{\hat{\alpha }}_{0,MLE},{\hat{\alpha }}_{1,MLE},{\hat{\alpha }}_{2,MLE},{\hat{\sigma }}_{t,MLE}^{2})$$ (30)

#### Conventional model

In the conventional model, we simply set $\sigma_{t}^{2}$ to be zero, and keep the rest to be the same for comparison.

### Model parameterization with Bayesian framework

We considered gene expression data from multiple perturbation conditions *p* = 1,…,*N*, with multiple time points *t* = 0, 15, 30, 60 min of each perturbation. This yields the probability to observe experimental data ***y***^(***obs***)^ as:

$$P(y^{\left(obs\right)})=\prod_{p}\prod_{i}P(y_{p,t_{i}}^{\left(obs\right)})$$ (31)

The statistical model of observing the experimental data ***y***^(***obs***)^ given its true value $y_{p,t_{i}}$ can be expanded as:

$$P(y^{\left(obs\right)})=\prod_{p}\prod_{i}I(y_{p,t_{i}}\leq 3)\Gamma ({y_{p,t_{i}}^{\left(obs\right)}|y}_{p,t_{i}},\sigma_{bg}^{2})+I(y_{p,t_{i}}>3)N(y_{p,t_{i}}^{\left(obs\right)}|y_{p,t_{i}},\sigma_{p,t_{i}}^{2})$$ (32)

#### Likelihood function

In our work, the experimental data ***y***^(***obs***)^ is explained by the combination of two parts, 1) model simulated $y_{p,t_{i}}$, which is determined by the GRS and kinetic parameters described by:

$$\theta =\left(\underset{GRS}{\underset{︸}{logic\;gate,K_{d1},K_{d2},K_{d3}}},\;\underset{kinetic\;parameters}{\underset{︸}{k_{syn},k_{proc},k_{0}}}\right)$$ (33)

2) data uncertainty, which is estimated from global trend ${\hat{\xi }}_{prior}=({\hat{\sigma }}_{bg}^{2},{\hat{\alpha }}_{0},{\hat{\alpha }}_{1},{\hat{\alpha }}_{2},{\hat{\sigma }}_{t}^{2})$ in time-value error model. Therefore, the likelihood can be written as:

$$L(\theta )=\prod_{p}\prod_{i}P(y_{p,t_{i}}^{\left(obs\right)}|y_{p,t_{i}}(\theta ),{\hat{\xi }}_{prior})=\prod_{p}\prod_{i}\left(I(y_{p,t_{i}}\leq 3)∙\Gamma (y_{p,t_{i}}^{\left(obs\right)}|y_{p,t_{i}}(\theta ),{\hat{\sigma }}_{bg}^{2})+I(y_{p,t_{i}}>3)∙N(y_{p,t_{i}}^{\left(obs\right)}|y_{p,t_{i}}(\theta ),{\hat{\alpha }}_{0}+{\hat{\alpha }}_{1}∙y_{p,t_{i}}(\theta )+{\hat{\alpha }}_{2}∙{y_{p,t_{i}}(\theta )}^{2}+{Slope}_{p,t_{i}}^{2}∙{\hat{\sigma }}_{t}^{2})\right)$$ (34)

We took the negative log likelihood for the optimization.

#### Computational analysis with data uncertainty

We define the distance between gene expression *i* and gene expression *j* as the average of *NLL*(*i*,*j*) and *NLL*(*j*,*i*), where *NLL*(*i*,*j*) is the negative log likelihood to observe gene expression *i*, given gene expression *j* as ground truth. We then performed hierarchical clustering with single linkage approach and the defined distance matrix.

#### Computational workflow implementation

We first applied time-value error model to estimate data uncertainty level. We took average of two replicates of TF activities as input. We then incorporate averaged TF activities as input, simulated gene expression data as output and their estimated data uncertainty into the model parameterization part.

#### Parameters estimation and model selection

To estimate parameters and select logic gates, we first optimized parameters for each logic gate, and picked the best fitted logic gate and its estimated parameters among all eight logic gates. In addition, we constrained the range of *K*_*d*_ from 10 fold weaker and stronger than the weak and strong regulation strength in the process of optimization (i.e. from 0.01 to 100), as *K*_*d*_ has minimal effect on the GRS output beyond this range. All the parameters are optimized in logarithmic scale, as it enables the algorithm to quickly search large space. We used multi-start local optimization approach, as it has been shown outperformed or at least perform as well as some global optimization methods [40]. Specifically, we employed the optim function with method Limited-memory BFGS (L-BFGS) in R for optimization, because it provides constrains for parameters, and it is computationally efficient. We used 300 randomly sampled initial sets of parameters for optimization for each logic gate. To ensure confidence that we sample the parameter space sufficiently, we examined the identifiability of each GRS as a function of the number of random initializations, and ensured it converges with 300 initial sets. The best fitted parameter set of 300 initial sets is chosen for the parameters of each logic gate. As all the logic gates have the same model complexity i.e. the same number of parameters, we simply select the logic gate based on their likelihood instead of using Akaike information criterion (AIC) or Bayesian information criterion (BIC). While our workflow allows people to select multiple good fitted models based their own criteria, we simply selected the logic gate with highest likelihood as the best fitted model.

#### Varying data uncertainty level

We tested our error model and pipeline with different level of data uncertainty. As each uncertainty sources don’t equally contribute to the final data uncertainty, we adjusted the altered level of each uncertainty source, so that the final error contribution from each of uncertainty sources would be approximately the same. After we simulated the data, we examined the overall uncertainty (mean of empirical estimated variance of all the points) caused by all the uncertainty sources (Table 4).

**Table 4:** Simulated uncertainty in multiple uncertainty levels.

Uncertainty Source	Form	Distribution	*θ*				
TF amplitude	*TF*∙(1+*ε*)	ε∼N(0,θ)	0.25x	0.5x	4.0 x 10^−4^	1.1x	1.45x
Gene response time	*t*+*ε*	ε∼N(0,θ)	0.25x	0.56x	25	2x	4x
Parameters (*K*_*d*_, *k*_*syn*_ *k_proc_*)	*Parameters*∙*e*^*ε*^	ε∼N(0,θ)	0.25x	0.5x	4.0 x 10^−4^	1.1x	1.45x
Parameters (*k*_0_)	*k*_0_+*ε*	*ε*~*U*(0,*θ*)	0.5x	0.75x	2.0 x 10^−2^	1.05x	1.2x
Sample handing time	*t*+*ε*	ε∼N(0,θ)	0.25x	0.56x	25	2x	4x
Sequencing error	*y*+*ε*	$\varepsilon ∼N(0,\theta ∙y^{2})$	0.25x	0.56x	1.0 x 10^−2^	1.1x	1.4x
Overall Uncertainty			0.21x	0.53x	1x	1.64x	2.66x

### Macrophage cell culture

Primary Bone Marrow Derived Macrophages (BMDMs) were prepared by culturing bone marrow cells from femurs of female 8–12 weeks old WT mice or different knock-out mice in L929-conditioned medium by standard methods [18]. BMDMs were grown for 7 days and stimulated with different agonists on day 8. BMDMs were stimulated with 100 ng/ml LipidA, 100 ng/mL LPS, as well as with a TLR2/1 agonist, the synthetic triacylated lipoprotein Pam3CSK4 (PAM) (3 μg/mL).

### TF activity quantification and normalization

Western blotting analysis and EMSAs were conducted with standard methods as described previously [18]. Briefly, nuclear extracts were prepared by hypotonic cell lysis and high salt extraction of nuclear proteins. The band intensities for Western blots or EMSA gels were measured in Image-Quant software. The samples within a timecourse that had peak intensities for different perturbations were run on the same gel, quantified and used for normalization of respective band intensities of different agonists.

To quantify the TF activity upon stimulation, we first linearly scaled the value of band intensities so that for each perturbation to the range that the basal and peak band intensity to be 1% and 100% by the formula:

$${TF}_{scaled}=0.99\frac{{TF}_{raw}{-TF}_{basal}}{{TF}_{peak}-{TF}_{basal}}+0.01$$ (35)

where *TF*_*scaled*_ and *TF*_*raw*_ are the values of TF band intensity before and after scaling. *TF*_*peak*_ and *TF*_*basal*_ are the peak and basal band intensity before scaling.

To compare TF activities in different perturbation conditions, we have normalized them by setting peak of wild type LipidA stimulation to be 1. We then normalized all the other perturbation conditions by multiplying each of the other perturbation conditions *p* with factor *p*_*norm*_:

$$p_{norm}=\frac{{TF}_{p}^{\left(peak\right)}}{{TF}_{WTLipidA}^{\left(peak\right)}}$$ (36)

where ${TF}_{p}^{\left(peak\right)}$ and ${TF}_{WTLipidA}^{\left(peak\right)}$ are the peak intensities of perturbation *p* and perturbation for wild type stimulated with LipidA that has been measured from the same gel.

#### Inference of MAPK-regulated transcription factor activity

We first defined the MAPK targeted genes: Egr1, Fos, and Dusp4, that are neither NFκB nor IRF targets. For those target genes, we estimate TF activities from their gene expression in RNA-seq. Specifically, for individual target genes, we linearly convert their gene expression (RPKM) so that in the WT stimulated with LipidA condition, the basal and peak value are 1% and 100%, by using the same formula described in the last section. Finally, we take the averaged gene expression from all the converted target genes as the inferred MAPK-regulated transcription factor activity.

### RNA-seq data processing

We have collected all the chromatin associated RNA-seq data from previously published work [19]. BAM files of chromatin associated RNA-seq data have been downloaded from GEO with the series accession number GSE67357. In the original BAM files, reads has been aligned to the mouse genome (NCBI37/mm9) with TopHat v1.3.3 by allowing up to two mismatches per read in one alignment. We followed the same standard in the paper to calculate RPKM for the chromatin associated RNA-seq by dividing all mapped reads within the transcription unit (both intron and exon) by the length of the entire locus.

Induced genes have been selected by the same criteria described in the paper [19]: the peak RPKM of induced genes is larger than 3 at any given time point, and the gene was induced more than 10 fold at any time point comparing to the time point 0min, with statistical significance p < 0.01 defined by the edgeR package in R Bioconductor [41]. In addition, a gene was also considered induced if it had more than a 5 fold gene induction at 15min.

For MAPK inhibited perturbation, we have collected both MAPK inhibited LipidA stimulation, and control condition (wild type in solvent with LipidA stimulation). To make the MAPK inhibited condition comparable to the other conditions, we normalized each time points by multiplying by the factor $\frac{{RNA}_{WT}(t_{i})}{{RNA}_{control}(t_{i})}$, where *RNA*_*WT*_(*t*_*i*_) and *RNA*_*control*_(*t*_*i*_) are the gene expression (RPKM) at time point *i* in wild type stimulated with LipidA and wild type with solvent stimulated with LipidA.

### ChIP-Seq data processing

We have collected all the RelA and IRF3 ChIP-Seq data from previously published work [19], and downloaded the bigWig files from GEO with the series accession number GSE67357. In the bigWig files, the reads had been aligned to the mouse genome (NCBI37/mm9) with Bowtie2. We examined and visualized the tracks of ChIP-seq data with Integrative Genomics Viewer (iGV) [42].

### Identify GRSs for immune response genes

#### Error quantification for chromatin associated RNA-seq data

We applied the developed error model to the collected chromatin associated RNA-seq data to estimate data uncertainty. For RNA-seq data with only single replicate (TRIF^-/-^ with LipidA stimulation, wild type with Pam3csk4 stimulation), we used the values estimated from wild type with LipidA stimulation.

#### Final model for studying immune response genes

We applied the same developed mechanistic model to identify GRSs for immune response genes. Specifically, to better capture basal gene expression, we set *k*_0_ = 0 for the model, and used experimental basal gene expression (gene expression in unstimulated condition, 0min) as gene basal expression for fitting, with formula:

$$y_{sim,p,i}{=y}_{simraw,p,i}+y_{basal,p}$$ (37)

where *y*_*sim*,*p*,*i*_ and *y*_*sim raw*,*p*,*i*_ are the final and raw model (with *k*_0_ = 0) simulated gene expression at time point *i* and perturbation condition *p*. y_basal,p_ is the experimental basal gene expression at perturbation *p*.

#### Fitting for experimental data

We applied the same Bayesian framework and likelihood function to identify GRS for immune response genes. After parameter optimization for all the 8 logic gates, we mapped them back to the 17 logics gates by assigning the 8 triple TF logics gates to single or dual logic gates if one or two TFs has null regulation strength (estimated *K*_*d*_ >100). Then, we only selected the potential GRSs for downstream analysis by setting a threshold on the fitting score (NLL <100).

## Supporting information

S1 Fig Exploring how logic gates behave as a function of variable TF regulation strengths. **Related to Fig 1.** Heatmaps of promoter activity as a function of logic gates (AND, OR) and varying TF1, TF2 regulation strengths.
(TIFF)

S2 Fig Extended details of GRS analysis. **Related to Fig 2.** Diagram explaining how the 216 possible GRSs were reduced to 93 potentially identifiable GRSs. First 69 poorly activated GRSs were removed (left side), as well as 54 redundant GRSs given that they are logically equivalent to a single TF (top right) or to two TFs with an AND gate (bottom right). This results in 93 potentially identifiable GRSs.
(TIFF)

S3 Fig Confirmation of conclusions with multiple alternative approaches. **Related to Fig 2.** (A). The heatmap as Fig 1G but reordered with different hierarchical clustering methods (complete and average linkage) in comparison to the used single linkage method used in Fig 1G. (B). The same plot as Fig 2C but only using average or complete linkage methods for hierarchical clustering. (C). Heatmap of gene expression in response to the 7 perturbation conditions from 1981 activatable GRSs that are generated by random sampling of all the parameters.
(TIFF)

S4 Fig Variance of each time points. **Related to Fig 3.** Comparison between directly estimated raw variance of two replicates and ground truth.
(TIFF)

S5 Fig Comparison between time-value error model and conventional model. **Related to Fig 4.** (A). Comparison of the estimated regulation strength between time-value error model, conventional error model, and raw variance with different levels of noise. (B). Confusion matrix of all the 93 estimated GRSs from time-value, conventional error models and raw variance.
(TIFF)

S6 Fig Extended study of perturbations and replicates trade-off. **Related to Fig 5** Comparison of the number of replicates and number of perturbation conditions for the identifiability of the 93 GRSs with a total of 8 datasets.
(TIFF)

S7 Fig Experimental data and identified GRS for lipid A induced genes. **Related to Fig 6.** (A). Graphs of measured and inferred TF activities in the perturbation conditions used by the caRNA-seq study. (B). Heatmap of measured nascent mRNA expression data together with fitness for all the candidate logic gates. The left side shows fitness (negative log likelihood) of all the possible logics. The white boxes are the logics that do not account for the data when mapping 8 logics back to the 17 logics. Genes are ordered by their expression with hieratical clustering approach. (C) Heatmaps of experimentally measured caRNA-seq time-course data and simulation data by the best-fit GRS models using the Time-Value error model or raw variance, as indicated. (D) Comparison of the negative log likelihood (NLL) of the best GRS model that contains any synergistic component to the negative log likelihood (NLL) of the best GRS model that does not contain any synergistic component.
(TIFF)
